# GIVE me your attention: Differentiating goal identification and goal execution components of the anti-saccade effect

**DOI:** 10.1371/journal.pone.0222710

**Published:** 2019-09-23

**Authors:** Owen Myles, Ben Grafton, Patrick Clarke, Colin MacLeod

**Affiliations:** 1 Centre for the Advancement of Research on Emotion, the University of Western Australia, Perth, Western Australia, Australia; 2 Curtin University, Bentley, Western Australia, Australia; University of Zurich, SWITZERLAND

## Abstract

The anti-saccade task is a commonly used method of assessing individual differences in cognitive control. It has been shown that a number of clinical disorders are characterised by increased anti-saccade cost. However, it remains unknown whether this reflects impaired goal identification or impaired goal execution, because, to date, no procedure has been developed to independently assess these two components of anti-saccade cost. The aim of the present study was to develop such an assessment task, which we term the Goal Identification Vs. Execution (GIVE) task. Fifty-one undergraduate students completed a conventional anti-saccade task, and our novel GIVE task. Our findings revealed that individual differences in anti-saccade goal identification costs and goal execution costs were uncorrelated, when assessed using the GIVE task, but both predicted unique variance in the conventional anti-saccade cost measure. These results confirm that the GIVE task is capable of independently assessing variation in the goal identification and goal execution components of the anti-saccade effect. We discuss how this newly introduced assessment procedure now can be employed to illuminate the specific basis of the increased anti-saccade cost that characterises various forms of clinical dysfunction.

## Introduction

Individuals differ in their cognitive control capability [1,2]. Investigators interested in such individual differences have developed a shared interest in a widely-used assessment approach known as the anti-saccade task. In this task, participants must initially attend to a central fixation cross, often displayed for varying temporal durations across successive trials. Next, a single visual object stimulus (e.g. a shape such as a solid oval) is presented to either the left or right side of the screen. Immediately upon its presentation, participants are required to make a saccadic response either towards or away from this visual object, depending on block condition. On “pro-condition” blocks, participants must execute a saccadic shift towards the visual object, whereas on “anti-condition” blocks, they must instead execute a saccadic shift away from the visual object, towards the opposite side of the screen. Making a saccadic response away from the visual object is challenging, and so participants are generally slower to make the required saccade in anti-condition blocks compared to pro-condition blocks, with the magnitude of this difference depending on the specific task parameters employed and the cognitive load carried by participants while performing the task [3–6]. This slowing of response times in the anti-condition compared to pro-condition is referred to as the anti-saccade cost.

It has repeatedly been demonstrated that this anti-saccade cost is increased in people with certain clinical disorders, including emotional disorders [7], neurological disorders [8], and developmental disorders [9]. For example, compared to healthy controls, individuals diagnosed with various forms of emotional dysfunction such as major depression [7] and anxiety [10,11], or suffering from neurological disorders such as Parkinson’s disease [8], or with developmental disorders such as autism spectrum disorders [9], exhibit heightened anti-saccade cost. Investigators have highlighted the potential applied importance of such findings, suggesting that the assessment of anti-saccade cost may help to improve diagnostic accuracy and/or serve as a marker of disease progression [12]. More important still, it has been argued that the cognitive anomalies that serve to increase anti-saccade cost in such cohorts may also contribute to their dysfunctional symptoms [13], making anti-saccade cost a candidate therapeutic target in cognitively focussed interventions.

Hence, there are good reasons for seeking to understand the cognitive basis of this anti-saccade cost effect. Many researchers argue that anti-saccade costs are lowest in those with the greater inhibitory attentional control [14], and so attribute elevated anti-saccade cost to impairments in inhibitory attentional control [15]. However, other investigators suggest alternative conceptions of the processes that underpin anti-saccade cost [16]. For example, some accounts are quite general in nature, such as the suggestion that variation in anti-saccade cost reflects individual differences in fluid intelligence or global processing speed [17,18]. Other accounts are more specific, such as the idea that performing an anti-saccade requires the time-consuming inversion of the pre-potent saccadic movement vector, in order to compute and control execution of the opposing saccadic movement vector [19].

Despite the differences between alternative accounts, investigators agree that performance on the anti-saccadic task must logically involve two key components processes [3–5]. Specifically, performing this task requires participants to: i. correctly identify whether to saccade to the right or to the left (i.e. saccadic goal identification); and ii. swiftly and successfully execute this saccadic goal (i.e. saccadic goal execution). In the anti-saccade task, as soon as the visual object appears, the participant must first identify whether their goal is to saccade to the left or to the right (which will depend on where this visual object appears, and whether the trial is given on the pro-condition or anti-condition). Then the participant must swiftly execute this saccadic goal, by making a saccade either to the left or to the right side as required. Thus, the slowing observed in the conventional anti-saccade task, on anti-condition relative to pro-condition blocks, could reflect a combination of two quite differing effects; slowing to formulate the correct saccadic goal, or slowing to implement this saccadic goal, in the former relative to the latter condition. It follows from this that elevations in anti-saccade cost could be driven by a deficit in anti-saccade goal identification, or a deficit in anti-saccade goal execution, or both.

However, our ability to differentiate these two candidate loci of variation in the magnitude of the anti-saccade effect has been hindered by the fact that current assessment approaches cannot yield independent sensitive measures of variation in anti-saccade goal identification and in anti-saccade goal execution components of anti-saccade task performance. Thus, it remains unknown whether previously observed clinically-linked elevations in anti-saccade cost result from inflated anti-saccade goal identification cost, from inflated anti-saccade goal execution cost, or both. The capacity to independently assess individual differences in these two facets of anti-saccade cost would enable investigators to more precisely pinpoint the locus of the elevated anti-saccade cost that characterize different clinical disorders. In addition to advancing understanding by illuminating underlying mechanisms, this may also yield applied benefits, by identifying the precise mechanism(s) that could most usefully be targeted in therapeutic interventions designed to enhance cognitive control in such cohorts.

The aim of the present study was to develop a novel assessment task capable of independently and sensitively assessing individual differences in anti-saccade goal identification and goal execution, which we term the Goal Identification Vs. Execution (GIVE) task. The GIVE task comprises two subtasks. One subtask, which we label the *Saccadic Goal Identification Subtask*, is designed to measure the speed with which participants can identify, when the visual object stimulus appears, whether the appropriate goal is to saccade to the left or to the right side. Similar to keyboard based versions of the anti-saccade task, such as that employed by Hunt & Klein [20] and others [5], or visual Simon tasks [21], participant responses depended on the position of the visual object. However, in this saccadic goal identification subtask, participants did not make a saccadic response, but kept their gaze fixed on the centre of the screen. Instead, they were required to respond only by indicating the direction in which they would move their eyes if they were completing the equivalent trial on the conventional anti-saccade task. This subtask permits assessment of the degree to which correct saccade goal identification is slowed in anti-condition blocks compared to pro-condition condition blocks, without the measure being contaminated by variation in speed of saccadic goal execution, as no saccade is executed in this subtask.

The other subtask, which we label the *Saccadic Goal Execution Subtask*, is designed to measure the speed with which participants can execute a predetermined saccadic goal (implementing a preceding instruction to saccade to the left or to the right), at the time point when the visual object appears, regardless of its location. By varying the object position, it is sometimes the case that the execution of this predetermined goal of saccading either left or right will involve the participants making a saccade towards the visual object (pro-condition), and sometimes it will involve making a saccade away from the visual object (anti-condition). Hence, this subtask permits assessment of the degree to which saccadic goal execution is slowed in anti-condition blocks compared to pro-condition condition blocks, without the measure being contaminated by variation is speed of formulating the goal of saccading left or right, as this saccadic goal is formulated prior to trial onset in this subtask.

We delivered the GIVE task, along with a conventional anti-saccade task, to a sample of undergraduate student participants. We first sought to determine whether our participant sample displayed the expected slowing of response latencies on anti-condition blocks, relative to pro-condition blocks, on the conventional anti-saccade task. We next went on to determine whether our participants displayed slowing of response latencies on anti-condition blocks, relative to pro-condition blocks, on the saccadic goal identification and/or on the saccadic goal execution subtasks of the GIVE. Finally, we went on to empirically evaluate: i. whether slowing observed on anti-condition blocks compared to pro-condition blocks, in the two subtasks of the GIVE, were independent of one another, and ii. whether slowing observed on anti-condition blocks compared to pro-condition blocks, in the two subtasks of the GIVE task, predicted independent variance in the anti-saccade cost effect observed on the conventional anti-saccade task.

## Method

### Participants

Fifty-one participants were recruited from the University of Western Australia undergraduate participant pool, and received course credit for their participation (M_age_ = 20.10, SD = 4.65, range = 17–23, 39 female). In a multiple regression with two predictor variables, this number of participants provides the capacity to detect effects that fall above *f*^2^ = .20, with a probability greater than .80, at an alpha level of .05 [22]. All participants had normal or corrected-to-normal vision at the time of completing the experiment.

### Apparatus

Task stimuli were presented on a 24ʺ widescreen LCD monitor. Participant eye-gaze was monitored using a desk mounted Eye-Link 1000 Plus eye-tracking system, running at 1000 Hz. Stimuli presentation was controlled using the Experiment Builder software package (SR Research Ltd, Mississauga, Canada).

### Assessment tasks

#### Conventional anti-saccade task

The present anti-saccade task was closely based on the approaches employed by Derakshan and colleagues [10,23]. Each trial commenced with the presentation of a fixation cross in the centre of the screen, for either 1000ms, 1500ms, or 2000ms, with equal probability. Participants were required to maintain continuous eye-gaze on this fixation cross in order for the fixation cross to disappear, and the trial to proceed. Following fixation cross offset, a single visual object stimulus was presented for 600 ms, positioned with equal frequency either 130mm to the left or right of screen centre. This visual object stimulus was a solid white oval (H = 63mm; W = 35mm). Immediately upon presentation of this oval stimulus, the participant was required to make a saccadic movement. The specific saccadic movement required was determined by instructions delivered prior to each block of trials. On pro-condition trial blocks, participants were required to shift their gaze left or right when the stimulus appeared left and right respectively (i.e. to make a saccade towards the oval stimulus). On anti-condition trial blocks, participants were required to shift their gaze right or left when the stimulus appeared left and right respectively (i.e. to make a saccade away from the oval stimulus). The response latency to successfully perform the required saccadic movement was recorded. The next trial commenced 500ms later. In total, six blocks of 12 trials were presented across the anti-saccade task. Half of the blocks were delivered in the pro-condition, and the other half were delivered in the anti-condition. These blocks of trials were delivered in a random order. It was expected that participants would be slower to make the required saccadic response on anti-condition blocks than on pro-condition blocks.

#### Goal Identification Vs. Execution (GIVE) task

**Saccadic Goal Identification Assessment Subtask**: This subtask was designed to assess individual differences in the relative speed to correctly identify whether the goal was to execute a saccadic to the left or to the right, on anti-condition trial blocks compared to pro-condition trial blocks, without the need to actually execute these saccadic goals. The physical and temporal characteristics of each trial were the same as in the conventional anti-saccade task. However, in this subtask, participants had to keep their eye-gaze fixed in the centre of the screen for the duration of the trial, and were required only to indicate the direction of the saccadic movement that would have been required had this trial been delivered in that condition of the conventional anti-saccade task (which they had just completed). On half the blocks, participants were told to indicate the direction that their gaze would have been required to move in the pro-condition of the conventional anti-saccade task. On the other half of the blocks, participants were told to indicate the direction that their gaze would have been required to move in the anti-condition of the conventional anti-saccade task. They responded using the keyboard, by pressing the left or right arrow button to indicate the direction of the saccade goal they identified as being the requirement. The latency and accuracy of this keyboard response was recorded. In total, six blocks of 12 trials were presented across this subtask, three blocks delivered in the pro-condition and three in the anti-condition trials. These blocks of trials were delivered in a random order. It was expected that participants would be slower to correctly identify the saccadic goal in anti-condition blocks, than to correctly identify the saccade goal in the pro-condition blocks.

**Saccadic Goal Execution Assessment Subtask**: This subtask was designed to assess individual differences in relative slowing to correctly execute the predetermined saccadic goal of making a saccade either to the left or right, when this happened to result in a saccade away from a presented oval stimulus (anti-condition blocks) or a saccade towards a presented oval stimulus (pro-condition blocks). Again, the physical and temporal characteristics of each trial were the same as those in the conventional anti-saccade task. The key methodological difference was that, in this subtask, prior to the commencement of each trial, participants were explicitly told whether their goal was to make a saccadic shift to the left or to the right as soon as the oval appeared (regardless of its position on screen). On half of the blocks, participants were instructed that their goal was to make a saccade to the left as soon as the oval appeared. On the other half of the blocks, they were instead instructed that their goal was to make a saccade to the right as soon as the oval appeared. On half of the trials within a block, the oval stimulus appeared in the opposite side of the screen to the location that participants had the goal of saccading towards (anti-condition trials). On the other half of the trials within a block, the oval stimulus appeared in the same location that participants had the goal of saccading towards (pro-condition trials). In total, six blocks of 12 trials were presented across this subtask, and these blocks of trials were delivered in a random order. Speed and accuracy of saccadic responses were recorded. It was expected that participants would be slower to correctly execute their predetermined goal of shifting their gaze to the left or right screen location on anti-condition trials than on pro-condition trials.

### Procedure

This study was approved by the UWA Human Research Ethics Committee (RA/4/1/5243). Participants provided written consent and were tested individually. To minimise head movements during eye-tracking, the participant was positioned in a head-rest affixed 60 cm from the computer monitor. The Eyelink 1000 Plus was calibrated for each new participant. Calibration was conducted using nine calibration points, and drift correction was performed prior to each block of trials. Participants were then given instructions for the conventional anti-saccade task, before completing two blocks of six practice trials (six pro-condition trials and six anti-condition trials), with corrective feedback. Upon completion of these practice trials, the assessment trials for the conventional anti-saccade task were delivered. Next, participants were given instructions for the GIVE task. Participants always completed one of the GIVE subtasks before the other, with the order of these subtasks counter-balanced across participants. Two blocks of six practice trials (six pro-condition trials and six anti-condition trials) of the upcoming subtask were always delivered prior to the participant completing the assessment trials of that subtask. Eye-tracker calibration was checked after each block of trials, and corrections to the calibration made as required. Upon completion of the GIVE task, participants were thanked for their participation and debriefed.

### Statistical methods

We first sought determine whether each of our sub-tasks demonstrated the slowing of response latencies usually observed on the conventional anti-saccade task. This was achieved by comparing response latencies on pro-condition trials with response latencies on anti-condition trials, using a series of within-subjects t-test. Next, we calculated an index of the anti-condition cost shown by each participant on each subtask, by subtracting their average response time for that task delivered in pro-condition from their average response time when the task was delivered in anti-condition. We proceeded to determine whether there was a correlation between the anti-condition cost indices on the two sub-tasks of the GIVE, before then conducting the planned multiple regression analysis, to determine whether independent variance in anti-saccade cost observed on the conventional anti-saccade was predicted by the two anti-condition cost measures provided by the GIVE task, respectively reflecting cost in terms of saccadic goal identification and cost in terms of saccadic goal execution.

## Results

One participant was excluded from data analysis due to difficulties in tracking their eye-movements. In keeping with data cleaning approach adopted by Derakshan et al [10], for each experimental task, only the first response made by the participant was included in the data analysis, and responses that were incorrect, anticipatory responses, or responses that occurred after the oval stimulus was cleared from the screen, were eliminated from analysis. Anticipatory responses were defined, using Derakshan et al.’s [10] criterion, as responses less than 83ms. Across the tasks, this led to the exclusion of 7.9% of responses. On the Anti-Saccade Goal Identification subtask, trials on which the participant failed to comply with the instruction to keep their eye-gaze fixed in the centre of the screen for the duration of the trial were eliminated. This led to the exclusion of 2.1% of responses.

We first sought to determine whether participants in our sample exhibited the expected slowing on anti-condition trials, relative to pro-condition trials, when performing the conventional anti-saccade task. Response times on both pro-condition trials and on anti-condition trials were found to demonstrate high reliability, indicated by Spearman-Brown split-half reliability co-efficients of .95, *p* < .001, and .97, *p* < .001, respectively. Mean response time was 178.45 ms (SD = 36.56) on pro-condition trials, and 255.71 ms (SD = 36.56), on anti-condition trials. A paired-samples t-test revealed that this slowing on anti-condition trials compared to pro-condition trials was statistically significant, *t* (49) = 17.15, *p* < .001, confirming that participants demonstrated the usual anti-saccade cost on this conventional anti-saccade task.

We then examined response times on each of the GIVE sub-tasks. In the saccadic goal identification sub-task, response times again demonstrated high reliability both in the pro-condition trials and in the anti-condition trials, demonstrated by Spearman-Brown split-half reliability coefficients of .93, p < .001, and .94, p < .001, respectively. Mean response time was 391.58 ms (SD = 44.37) on pro-condition trials, and 412.27 ms (SD = 41.70) on anti-condition trials. This difference in means was shown to be statistically significant, using a paired-samples t-test, *t* (49) = 5.33, *p* < .001. Thus, participants were generally slower to correctly identify whether their goal was to shift their gaze to the left or right, when this saccadic goal identification subtask was delivered in the anti-condition than when it was delivered in the pro-condition.

Next, response times obtained on the saccadic goal execution subtask of the GIVE were examined. Once again, the response times demonstrated high reliability both in the pro-condition trials and the anti-condition trials, with Spearman-Brown split-half reliability coefficients of .93. *p* < 0.001, and .95, *p* < 0.001, respectively. Mean response time on this subtask was 175.62 ms (SD = 25.97) in the pro-condition, and 220.75 ms (SD = 30.84), in the anti-condition, with a paired-samples t-test confirming that this represents significantly slower responding on anti-condition trials than on pro-condition trials, *t* (49) = 18.87, *p* < .001. This indicates that participants were relatively slower to execute the predetermined goal of making a saccadic to the right or left, when such saccadic goal execution required making a saccade away from the oval stimulus rather than towards it.

Together, these findings confirm that participants exhibited the expected patterns of response slowing, in the anti-condition compared to the pro-condition, not only on the conventional anti-saccade task, but also on both sub-tasks of the GIVE, respectively assessing saccadic goal identification and saccadic goal execution. We went on to determine whether individual differences in the magnitude of this response slowing in the anti-condition compared to the pro-condition were independent across the two GIVE subtasks. To do this, we computed an index of the anti-condition cost not only on the conventional anti-saccade task (anti-saccade cost; Spearman-Brown split-half reliability = .94), but also on the saccadic goal identification sub-task of the GIVE (anti-saccadic goal identification cost; Spearman-Brown split-half reliability = .67) and on the saccadic goal execution sub-task of the GIVE (anti-saccadic goal execution cost; Spearman-Brown split-half reliability = .62), as described in the Method section. Pearson’s correlation analysis revealed no evidence of an association between these two index scores, *r* = -.03, *p* = .84, suggesting that these two GIVE sub-task cost indices vary independently of each other. This conclusion was supported by the outcome of a Bayesian correlation. The resulting Bayes Factor of BF_01_ = 5.56 represents substantial support for the null hypothesis [24], and lends further weight to the independence of the two cost indices provided by the GIVE task.

**Fig 1:**
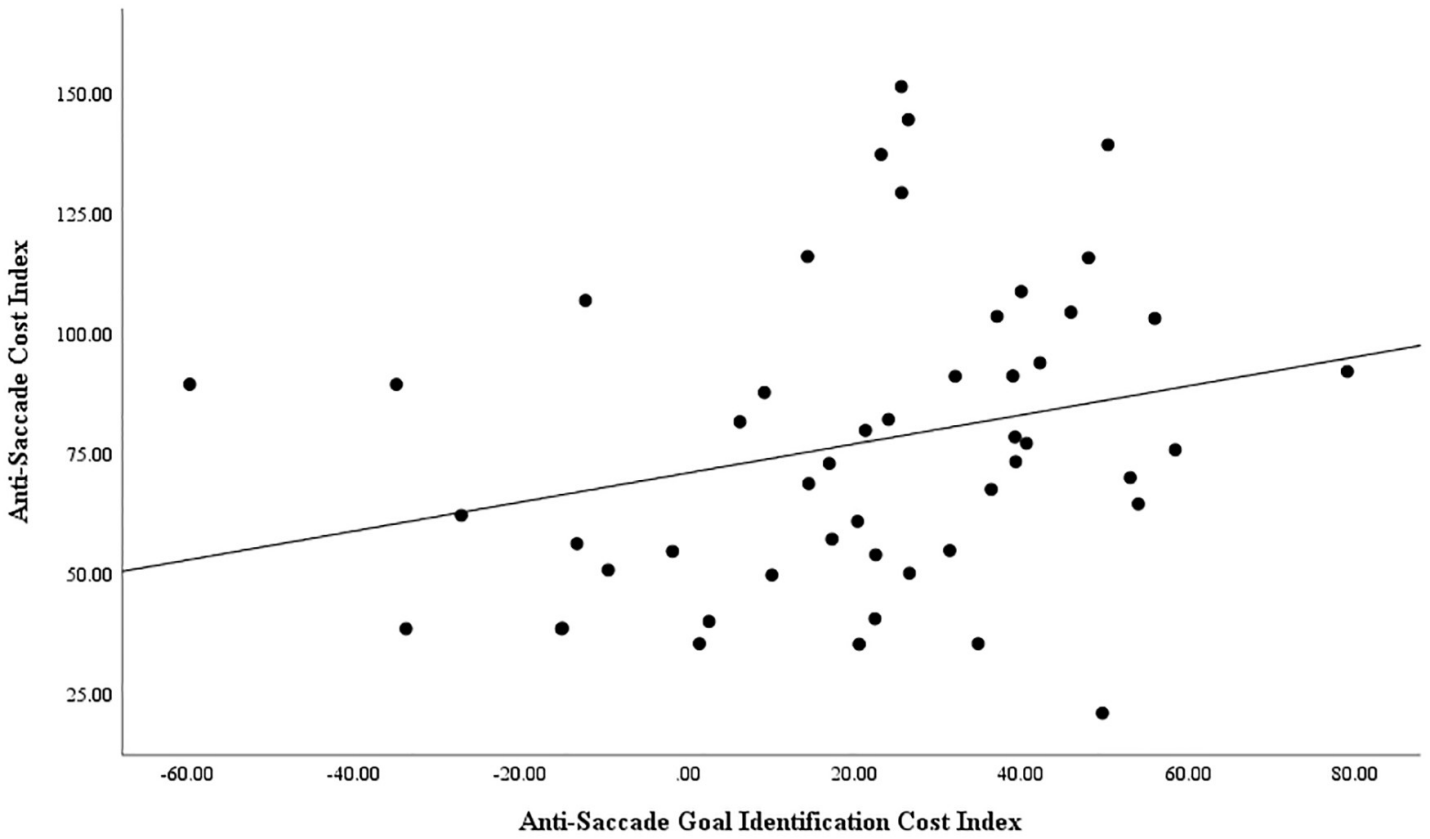
Scatterplot of association between anti-saccade goal identification cost index and conventional anti-saccade cost index, showing line of best fit.

**Fig 2:**
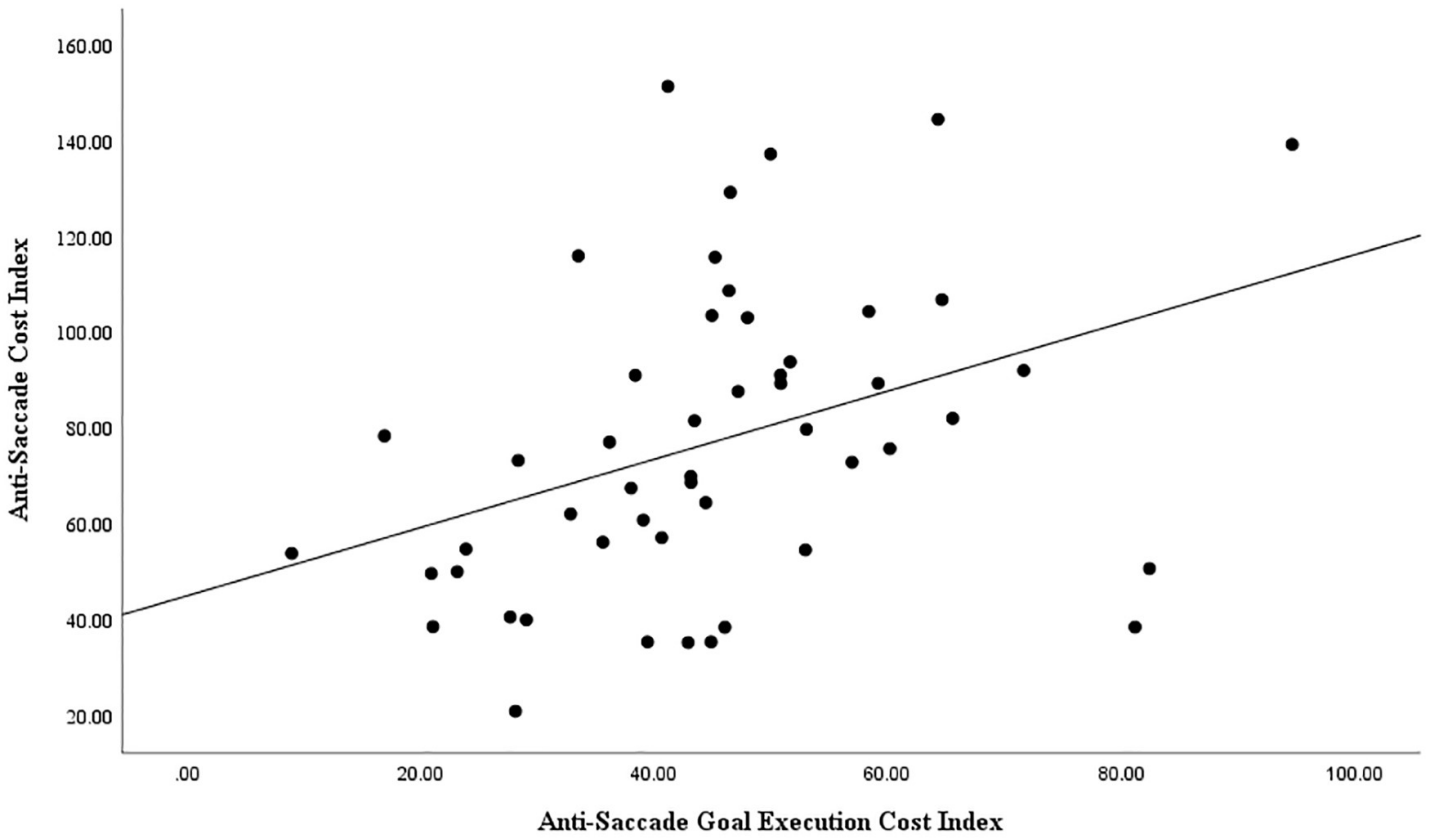
Scatterplot of association between anti-saccade goal execution cost index and conventional anti-saccade cost index, showing line of best fit.

As planned, we went on to examine whether variation in each of these two cost indices yielded by the GIVE, respectively reflecting anti-saccadic goal identification cost and anti-saccade goal execution cost, accounted for independent variance in the anti-saccade cost observed on the conventional anti-saccade task. Specifically, the anti-saccade cost index scores obtained using the conventional anti-saccade task scores were entered as the dependent variable in a regression analysis, with the anti-saccadic goal identification cost index scores and anti-saccadic goal execution cost index scores obtained using the GIVE sub-tasks simultaneously entered as independent variables. The overall model was significant, *F* (2, 49) = 6.53, *p* = .003, *R*^*2*^ = .22. This indicates the overall model accounted for 22% of the variance in anti-saccade cost. Moreover, the GIVE task anti-saccade goal identification cost index scores, *t* (49) = 2.11, *p* = .040, β = .272, and the GIVE task anti-saccade goal execution cost index scores, *t* (49) = 3.00, *p* = .004, β = .387, each did indeed predict independent variance in the anti-saccade cost index scores obtained using the conventional anti-saccade task, accounting for 7% and 15% of the variance in anti-saccade cost respectively. The relationship between anti-saccade cost on the conventional anti-saccade task and the GIVE task measures of anti-saccade goal identification cost and anti-saccade goal execution cost is shown in Figs 1 and 2 respectively. This pattern of results supports the capacity of the GIVE tasks to measure independent variance in the saccadic goal identification component and in the saccadic goal execution component of anti-saccade task performance. Furthermore, this demonstrates that individual differences in both component processes assessed by the GIVE contribute to variation in the anti-saccade cost effect commonly observed on the conventional anti-saccade task.

It is possible that the cost indices yielded by the conventional anti-saccade task, and by the two GIVE sub-tasks, might all be greater in people who have slower overall processing speed, and such differences in processing speed could potentially drive the observed association between these cost measures. In order to address this possibility, we carried out mediation analyses to determine whether the association between the anti-saccade cost index scores, and each of the GIVE sub-task cost index scores, reflected the mediating influence of overall response speed, as indexed by average RT latency across all trial types. The outcome of these analyses revealed that the association between the anti-saccade cost index scores and the anti-saccade goal identification cost index scores was not significantly mediated by overall processing speed (Sobel test = .977, p = .33, bootstrap confidence interval = -.11 to .13). Bootstrap confidence intervals of the indirect effect were obtained using 5000 bootstrap samples. Further, the association between the anti-saccade cost index and the anti-saccade goal execution cost index also was not significantly mediated by overall processing speed (Sobel test = -.36, p = .72. bootstrap confidence interval^3^ = -.49 to .30). Thus, this pattern of findings suggest that the observed association between these cost measures is not driven by individual differences in overall processing speed.

## Discussion

The aim of this study was to develop and evaluate a new assessment approach, capable of independently assessing variation in saccadic goal identification and saccadic goal execution. Our findings show that the measures of anti-saccade goal identification cost and anti-saccade goal execution cost yielded by our newly developed GIVE task were uncorrelated with one another, and Bayesian analysis further supports the conclusion that no such association exists. The absence of an association between these two cost indices cannot be attributed to the insensitivity of one or other measure, perhaps reflecting reduced sensitivity when a key press response or eye-tracking is used to compute the cost index. Such an explanation is incompatible with the observation that each cost measure yielded by the GIVE task accounted for statistically significant independent variance in the anti-saccade cost effect observed on the conventional anti-saccadic task. Thus, taken together, this pattern of findings suggests that the GIVE task is capable of differentiating and sensitively assessing the individual differences in saccadic goal identification, and in saccadic goal execution, that independently contribute to anti-saccade cost in the widely-used anti-saccade task. This discussion will briefly consider the implications of the present demonstration that variability in goal identification and goal execution separately contribute to variation in anti-saccade cost, before highlighting the potential value of the GIVE task in pinpointing the loci of the elevated anti-saccade costs known to characterize a number of clinical conditions.

It will be important to ensure that the present pattern of findings can be replicated by other investigators. In part, this is likely to depend on the reliability of the GIVE task. While the present study demonstrates that response latencies and index measures on both GIVE sub-tasks are characterized by acceptable internal reliability for cognitive tasks [25], determining their test-retest reliability will require appropriate extensions of the present work, that employ multiple assessment sessions. Should future research confirm the replicability of the presently findings, then it is reasonable to suppose that understanding of the conventional anti-saccade effect will be enhanced by illuminating the basis of variation in saccadic goal identification and saccadic goal execution. It seems likely that each of these two component processes will themselves reflect the operation of subsidiary cognitive mechanisms that, in time, can be empirically distinguished and independently assessed. For example, it seems reasonable to suppose that the process of saccadic goal identification within the anti-saccade task could be further broken down into the sub-components of coding the location of the visual object stimulus, accessing the task instruction for that trial block (concerning whether to shift gaze towards or away from the displayed object), and computing from these two sources of information whether the correct saccadic goal on the current trial is to saccade left or saccade right. Hence, individual differences in the saccadic goal identification component of the anti-saccade cost effect could potentially reflect the independent contributions of variability in each of these subsidiary processes. We encourage future researchers to continue the conceptual delineation of such component processes, while also developing novel assessment methodologies that can differentially assess these components, in order to construct a comprehensive understanding of the processes that contribute to variability in anti-saccade cost. For the moment, by enabling the discrete assessment of individual differences in saccadic goal identification and in saccadic goal execution, the newly developed GIVE task provides researchers with the capacity to illuminate the basis of the elevations in anti-saccade cost observed across differing clinical conditions.

Although there are a wealth of previous findings demonstrating increased anti-saccade cost in people with certain clinical conditions, the limitations of previous assessment approaches have made it impossible to determine whether this reflects a deficit in anti-saccadic goal identification, a deficit in anti-saccadic goal execution, or both. For example, the increased anti-saccade cost evident in people with Parkinson’s disease [8] could reflect a deficit in anti-saccade goal identification due to the cognitive restrictions evident in this disease [26], or may result from a deficit in anti-saccade goal execution due to the degeneration of oculomotor control that accompanies the disease [27]. By employing the GIVE task in future research studies to assess anti-saccade cost in these populations, and in other populations also characterized by increased anti-saccade cost (e.g. individuals with autism spectrum disorders, anxiety & elderly populations [7,9,10,28], it will become possible to identify which of these component processes is compromised in each of these conditions. Interventions designed to attenuate or reverse such deterioration in cognitive control then can target the precise mechanisms that underpins the particular problems experienced by each population of interest.

Future research of the type described will serve to pinpoint the loci of the elevated anti-saccade costs known to characterize different clinical conditions. But, for the moment, the work reported in this paper has given rise to a new assessment approach that can sensitively assess the individual differences in saccadic goal identification, and in saccadic goal execution, that independently contribute to variation in the anti-saccade cost effect observed on the conventional anti-saccadic task. We hope that the GIVE task will now enable researchers to refine our knowledge of the mechanisms that underpin increased the anti-saccade cost evident across a number of important clinical disorders, in ways that will advance understanding and inform intervention approaches.
